# Class B β-arrestin2-dependent CCR5 signalosome retention with natural antibodies to CCR5

**DOI:** 10.1038/srep39382

**Published:** 2016-12-23

**Authors:** Assunta Venuti, Claudia Pastori, Rosamaria Pennisi, Agostino Riva, Maria Teresa Sciortino, Lucia Lopalco

**Affiliations:** 1 Division of Immunology, Transplantation and Infectious Diseases, San Raffaele Scientific Institute, Milan, 20132, Italy; 2 Department of Chemical Biological Pharmaceutical and Environmental Sciences, University of Messina, Messina, 98166, Italy; 3 Third Division of Infectious Diseases, Luigi Sacco Hospital, University of Milan, Milan, 20157, Italy

## Abstract

CCR5 stimulation with natural ligands, such as RANTES, classically induces short-term internalization with transient activation of β-arrestins and rapidly recycling on the cell surface. Here we discovered that, in T cells, natural CCR5 antibodies induce a CCR5-negative phenotype with the involvement of β-arrestin2, which leads to the formation of a stable CCR5 signalosome with both β-arrestin2 and ERK1. The activation of β-arrestin2 is necessary to CCR5 signaling for the signalosome formation and stabilization. When all stimuli were washed out, β-arrestin1 silencing favors the activity of β-arrestin2 for the CCR5 signalosome retention. Interestingly, CCR5 turn from Class A trafficking pattern, normally used for its internalization with natural modulating molecules (i.e. RANTES), into a long lasting Class B type specifically induced by stimulation with natural anti-CCR5 antibodies. This new CCR5 pathway is relevant not only to study in depth the molecular basis of all pathologies where CCR5 is involved but also to generate new antidody-based therapeutics.

The seven-transmembrane receptors (7TMRs, also called G protein-coupled receptors, GPCRs) are the largest known group of integral membrane receptor proteins with important physiological and pathological functions.

Despite it being previously asserted that GPCRs are able to bind only their related G proteins, it is now well documented that they can bind to a large number of accessory proteins that influence and/or mediate various aspects of receptor function^1^,^2^,^3^,^4^,^5^.

Even more recently, the identification of β-arrestins as a scaffold for the assembly of a complex containing the receptor and effectors clearly demonstrates that they participate in signal transduction of many GPCRs^1^,^6^. In particular, the conformational changes induced by GPCRs in β-arrestins, especially when they assemble with the phosphorylated domains of the receptor, could lead to the transmission of signals to downstream kinases, such as mitogen-activated-protein kinases (MAPKs), Raf, MEK1, phosphoinositol-3-kinase (PI3K), Akt^7^,^8^,^9^,^10^. These modifications include: (i) phosphorylation, (ii) ubiquitination, (iii) SUMOylation, (iv) S-nitrosylation, and (v) acetylation^11^.

The ubiquitination of β-arrestin especially promotes its interaction with proteins that mediate endocytosis (e.g., clathrin) and signaling (e.g., ERK1/2), influencing spatial and temporal resolution of the complex^3^,^5^,^12^,^13^,^14^. Notably, 7TMRs can be functionally divided into two general categories based on the stability of interaction with β-arrestins following agonist activation: (i) “Class A” receptors, such as β2 adrenergic receptor (β2AR), form transient complexes with β-arrestin, show its transient ubiquination with weak induction of ERK1/2 activity; in contrast (ii) “Class B” receptors, including vasopressin V2 receptor (V_2_R), form tight receptor- β-arrestin complexes, mediated by its sustained ubiquitination and a strong ERK1/2 activity that is concentrated on endosomes. The endosomes containing complexes between activated 7TMR, activated and ubiquitinated β-arrestin and phosphorilated ERK are named as “signalosome”^1^,^15^.

The CC chemokine receptor 5 (CCR5), belonging to the GPCRs family, regulates trafficking and effector functions of immune cells^16^, but also is the main coreceptor of HIV, in association with CD4^17^.

Natural human antibodies, recognizing an epitope within the first extramembrane loop of CCR5 (ECL1), found in Long Term HIV infected subjects induce a long-lasting internalization (48 h) of the receptor, instead of the short-term kinetics described for other CCR5 ligands (60′–90′)^18^,^19^, and provide HIV protection^20^. Recently, we reported an hitherto unrecognized mechanism of CCR5 modulation mediated by G-protein-dependent ERK1 activity upon stimulation with anti CCR5 natural antibodies^21^, that promotes cytoplasmatic retention of phosphorylated ERK1.

Understanding if the long lasting CCR5 internalization, mediated by anti CCR5 antibodies, occurs through β-arrestin-dependent ERK1 activation could reveal new aspects about the mechanism that induce an ERK1-mediated CCR5-negative phenotype.

The present study is aiming to comprehensively investigate in depth the role of β-arrestin1/2 in the regulation of the receptor internalization ERK-mediated mechanisms and CCR5 re-expression on the cell membrane.

## Results

### Anti-CCR5 Abs induce the generation of the CCR5 signalosome with both β-arrestin1/2 and ERK1

**Figure 1:**
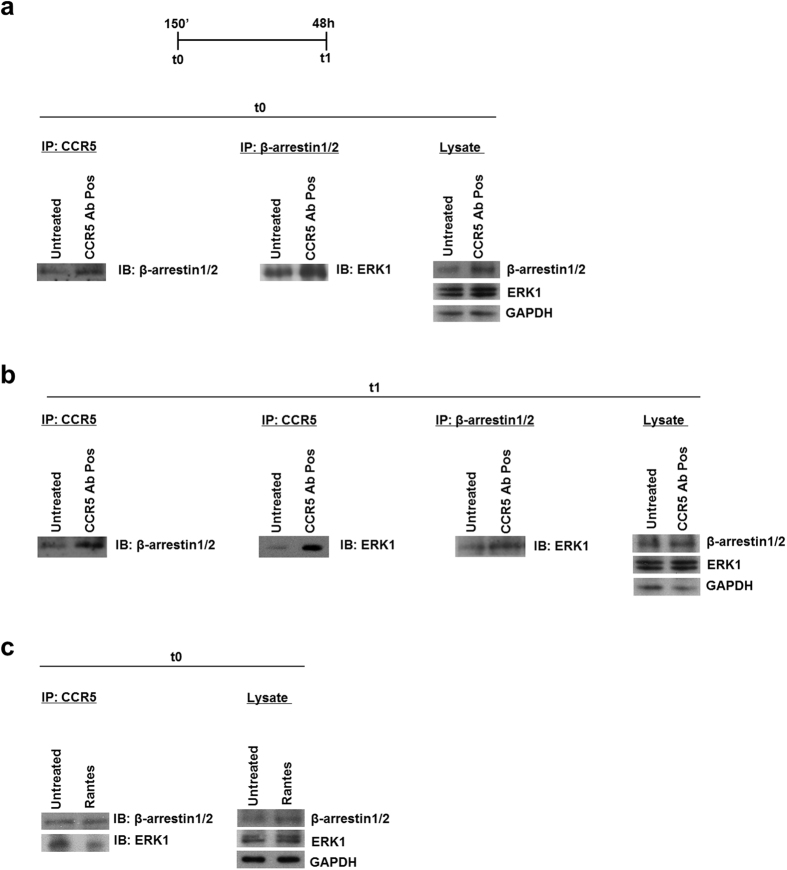
CCR5 interacts with both ERK1 and β-arrestin1/2. (**a–c**) Immunoprecipitation (IP) and immunoblot analysis (IB) of the CCR5 interaction with ERK1 and β-arrestin1/2 in R5**-**SupT1-M10 cells, which were treated or not with CCR5 Ab Pos and harvested at t0 (**a**) and t1 (**b**). In panel c, the cell were treated with Rantes and harvested at t0. Immunoblot analysis of total cell lysates was used as a control (right panels). Data are representative of three independent experiments.

Our group has recently demonstrated that CCR5 specific antibodies (CCR5 Ab Pos), found in Long Term HIV infected subjects (LTNPs), induce a long lasting internalization of the receptor (48 h) mediated by G-protein-dependent ERK1 activity, both in R5-SupT1-L23 and in CD4+ T lymphocytes^21^. Instead, other groups have demonstrated that when CCR5 internalization is mediated by RANTES, the level of receptor on the cell surface recovered to nearly 100% after 120′ incubation^4^,^22^,^23^. Taking into account that anti-CCR5 Abs lead to a statistically significant accumulation of β-arrestin1/2 as well^21^, we performed an immunoprecipitation assay to evaluate the interaction among the components and the temporal distribution of the CCR5 signalosome. R5-SupT1-M10 cell line, which displays medium levels of CCR5 expression^24^, was used to immunoprecipitate both CCR5 and β-arrestin1/2. We used a pool of 5 LTNPs sera, each containing CCR5 antibodies, or not (named CCR5 Ab Neg and used as a negative control), as previously described and characterized by our group^21^,^25^; we also used the chemokine RANTES, as a positive control^26^. In detail, the cells were incubated for 30′ with Rantes and with CCR5 Ab Pos or not, washed and incubated for a further 120′ (i.e., incubation for this group totaled 150′), t0, and for 48 h (t1); these two time points have been chosen on the basis of our previous findings^21^; briefly, 150′ corresponds to the initial internalization of the receptor and 48 h is required to achieve complete CCR5 internalization. The cells were then lysed and incubated either with CCR5- or β-arrestin1/2-antibody-pre-adsorbed beads, as described in Methods. Western blotting analysis performed after overnight incubation demonstrated that β-arrestin1/2 proteins interacted with both the CCR5 receptor and ERK1 protein in both untreated and treated cells. Figure 1a shows a specific induction of β-arrestin1/2 and ERK1, respectively, with CCR5 Ab Pos stimulation at t0; this complex was stable maintaining into the cells at least 48 h, as demonstrated by immunoblot at t1 (Fig. 1b). As expected, when the cells were treated with Rantes, the immunoblot at t0 showed a weaker β-arrestin1/2 and ERK1 binding (Fig. 1c), highlighting a reduction in the complex formation. This finding suggests that β-arrestin1/2 and ERK1 proteins are important components of the CCR5 signalosome upon stimulation with anti CCR5 Ab Pos and the interaction among them remains stable at least up to 48 h.

### Specificity of the role played by the two β-arrestins in the formation of the CCR5 signalosome

**Figure 2:**
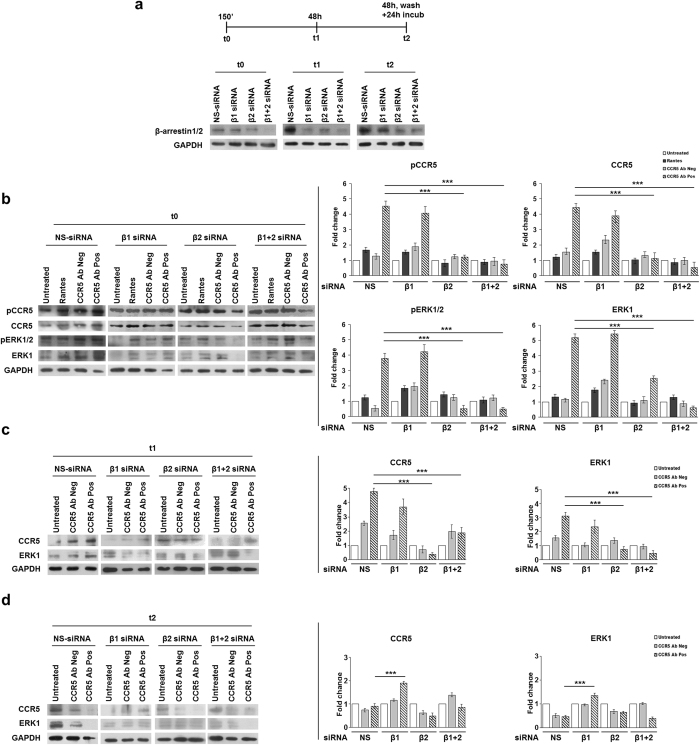
Role of specific siRNAs for β-arrestin1/2 in CCR5 regulation. (**a**) Schematic representation of kinetics experiment and immunoblot analysis of β-arrestin1/2 and GAPDH (loading control throughout) in whole R5-SupT1-L23 cell-lysates nucleofected with siRNAs directed to β-arrestin1 (β1), β-arrestin2 (β2) or both and with NS-siRNA (negative control) for all time points. (**b–d**) Immunoblot analysis of phosphorylated and total CCR5 and ERK1 in whole-cell lysates harvested after treatment with anti CCR5 Ab Pos and appropriate controls (RANTES and CCR5 Ab Neg), at t0 (corresponding to 30′ of CCR5 modulating factors incubation, washing and additional 120′ of cells incubation in medium without stimuli (150′)) (**b**), t1 (corresponding to 48 h of factors incubation) (**c**), t2 (corresponding to 48 h of factors incubation, washing and a further 24 h of cells incubation in medium without stimuli) (**d**). Protein levels have been determined with densitometric analysis of the blot with the T.I.N.A. program and expressed as fold change over the appropriate housekeeping genes. Bar graphs represented mean ± SD of three independent experiments. Student’s t-test was performed and p values are shown. *p ≤ 0.05, **p ≤ 0.01, ***p ≤ 0.001 (b,c right panels).

Based on these first results, we tested the precise role of β-arrestin1/2 in the CCR5 signalosome formation upon stimulation with anti-CCR5 Abs. In fact, it is well known that GPCRs can activate ERK1/2 proteins via β-arrestin-dependent pathways, with consequent retention of ERK1/2 proteins into the cytosol^27^,^28^,^29^,^30^,^31^. We analyzed a time course of anti-CCR5 Abs-induced signaling pathway after depleting cellular levels of β-arrestin1 or -2 or both by nucleofecting siRNAs specific to each isoform. The experiments were performed in the T lymphoblastoid R5-SupT1-L23 cell line, which has a CCR5 cell surface level and an activation status comparable to human CD4+ T lymphocytes^21^,^24^. The activation status of the cells has been evaluated by flow cytometry and the mean fluorescence (MFI) obtained with anti-CD38 antibody was 54.5 and 34.5 respectively, as previously reported^21^. To this end, 5 h post nucleofection, the cells were stimulated with CCR5 Ab Pos and their controls and then collected at t0 (150′: 30′ incubation, wash, further 120′ incubation), t1 (48 h), t2 (48 h, wash, further 24 h incubation) as described in Fig. 2a. Western blotting analysis showed that β-arrestin1/2 siRNAs duplexes, already tested by Shenoy *et al*.^15^ specifically decrease protein expression levels (Fig. 2a). Accordingly with nucleofection principle (AMAXA, Lonza), the knock down of genes analyzed was maximum within the first 48 h post nucleofection and the expression levels returned normal within 72–96 hours.

At t0, when the cells were nucleofected with non-targeting control siRNA (NS-siRNA), CCR5 Abs Pos triggered the phosphorylation with consequent accumulation of CCR5 and ERK1 proteins and the β-arrestin1 silencing had a non-significant effect on CCR5 and ERK1 activation (Fig. 2b); in contrast, the silencing of β-arrestin2 and of both β-arrestins, 1 and 2, showed a statistically significant reduction in phosphorylated and non-phosphorylated forms of both CCR5 and ERK1 (p ≤ 0.001), in the cells stimulated with CCR5 Ab Pos compared to the relative non-targeting control siRNA (NS-siRNA) (Fig. 2b), thus confirming that both β-arrestin2 and ERK1 play a major role in the formation of the CCR5 signalosome as demonstrated with the immunoprecipitation assays shown in Fig. 1a,b.

At t1, when the cells were nucleofected with both NS- and β-arrestin1 siRNAs and then incubated with CCR5 Ab Pos showed a greater accumulation of CCR5 and ERK1 proteins in comparison with CCR5 Ab Neg treated cells (Fig. 2c). On the other hand, the silencing of β-arrestin2 and of both β-arrestins, 1 and 2, had strongly reduced this cytosolic accumulation of CCR5 and ERK1 (p ≤ 0.001) under CCR5 Ab Pos stimulation.

Surprisingly, at t2, when all stimuli were washed out and replaced with fresh complete medium for an additional 24 h, CCR5 Ab Pos led to a statistically significant accumulation (p ≤ 0.001) of both CCR5 and ERK1 proteins only when β-arrestin1 was silenced.

These results clearly show that the CCR5 internalization, mediated by CCR5 Ab Pos, recruits ERK1 with the intervention of β-arrestins; in particular, taken together the data suggest an important role of β-arrestin2 in the CCR5 accumulation both during early events, in the signalosome formation (t0), and also when the internalization reached the maximum levels, thus acting for the stabilization of the CCR5 signalosome (t1). When the internalization is complete and the effect of the CCR5 Ab Pos is abrogated (t2), β-arrestin1 silencing affects the accumulation of the involved proteins, thus suggesting that the two β-arrestins could act in a different way within the CCR5 internalization pathway.

### β-arrestin2, but not ERK1, acts as an early stimulator of CCR5 gene expressions pathway

**Figure 3:**
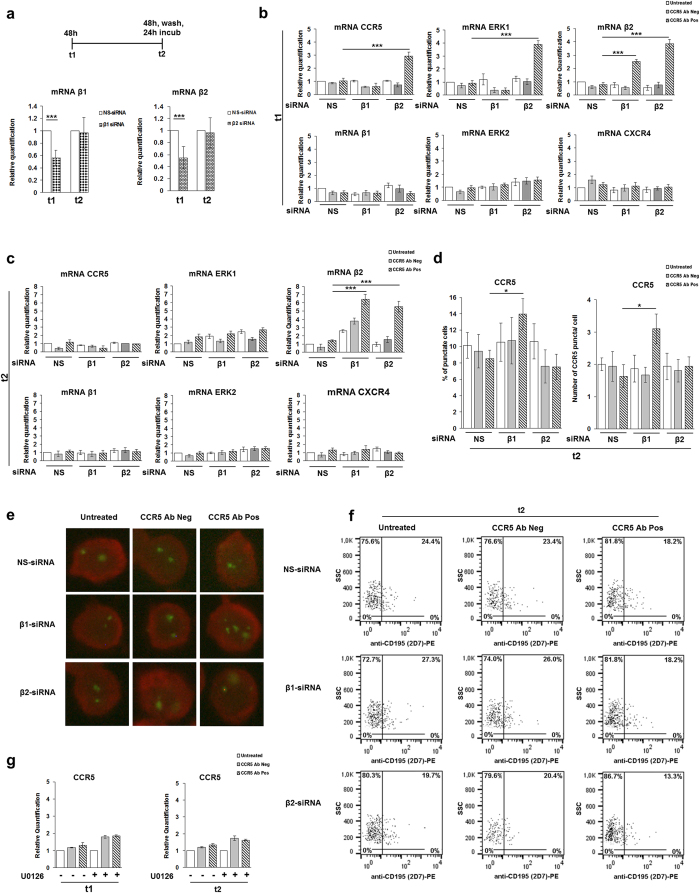
siRNAs specific for β-arrestin1/2, but not U0126 treatment, influence the CCR5 gene expressions pathway. (**a**) Schematic representation of kinetics experiment and real time RT-PCR analysis of β-arrestin1 and 2 genes expression in R5-SupT1-L23 cells nucleofected with siRNAs directed to β-arrestin1 (β1), β-arrestin2 (β2), compared with NS-siRNA. (**b,c**) Real time RT-PCR analysis of CCR5, ERK1, ERK2, β-arrestin2 (β2), β-arrestin1 (β1) and CXCR4 in the cells harvested after treatment either with anti CCR5 Ab Pos or CCR5 Ab Neg at t1 (corresponding to 48 h of factors incubation) (**b**), at t2 (corresponding to 48 h of factors incubation, washing and a further 24 h of cells incubation in medium without stimuli), compared with untreated cells of the control group (**c**). (**d**) The percentage of cells with the punctate form of CCR5 (left) and the number of CCR5 puncta per cell (right) in untreated and treated cells with antibodies (CCR5 Ab Pos and CCR5 Ab Neg) in siRNAs nucleofected R5-SupT1-L23 cells, stained with anti-CKR5(D6), are reported. (**e**) A representative immunofluorescence image of CCR5-positive cells. Evans Blue dye was used as a counter stain. (**f**) CCR5 cell surface expression (measured by flow cytometry with specific Mab to CCR5 (CD195)) in R5-SupT1-L23 cells nucleofected with siRNAs against β-arrestin1 (β1) and β-arrestin2 (β2) stimulated or not with CCR5 Ab Pos and CCR5 Ab Neg, at t2. Data are representative of three independent experiments. (**g**) Real time RT-PCR analysis of CCR5 in cells pre-treated, or not, with the U0126 (MAPKs inhibitor) for 1 h and stimulated with CCR5 Ab Pos and the appropriate control, compared to the untreated cells at t1 (left) and t2 (right). In Panels a–d, g, bar graphs represented mean ± SD of three independent experiments. Student’s t-test was performed and p values are shown. *p ≤ 0.05, **p ≤ 0.01, ***p ≤ 0.001.

In order to better define the role of the individual β-arrestins in the CCR5 modulation under anti-CCR5 Abs, in a parallel experiment accomplished in R5-SupT1-L23 cells, we examined the transcript levels of genes involved in the CCR5 pathway. Measuring with real time RT-PCR, β-arrestin1 and β-arrestin2 were statistically significantly silenced for 44% ± 12.1% and 45.5% ± 18.9% at t1, respectively; the expression of β-arrestins both 1 and 2 returned to basal level when the cells were interfered by siRNAs for more than 72 h (t2), as expected (Fig. 3a).

The down-regulation of β-arrestin2 underlined a statistically significant increase in CCR5 and ERK1 mRNAs only in the samples treated with CCR5 Ab Pos compared to the negative control siRNA (p ≤ 0.001 for both evaluation) at t1 (Fig. 3b), suggesting that the absence of β-arrestin2 could act as an early stimulator to the *de novo* synthesis of the proteins involved in the CCR5 signalosome.

Surprisingly, also the expression levels of β-arrestin2 were statistically significantly up-regulated (p ≤ 0.001), specifically upon CCR5 Ab Pos stimulation, not only when β-arrestin1 was silenced but also in the presence of β-arrestin2 depletion. On the other hand, even though the depletion of β-arrestin1 showed no effects on positive regulation of CCR5 and ERK1 genes synthesis, a statistically significant *de novo* synthesis of β-arrestin2 was evident at t1 in the cells treated with CCR5 Ab Pos compared to the relative negative control siRNA treated ones (p ≤ 0.001) (Fig. 3b); thus suggesting a gene expression switching from β-arrestin1 to β-arrestin2 synthesis, in the cells interfered with siRNA against β-arrestin1. Interestingly, at t2, a statistically significant genes expression was shown with β-arrestin2 only, in the samples silenced with siRNAs against both β-arrestin1 and β-arrestin2 (p ≤ 0.001), upon CCR5 Ab Pos stimulation (Fig. 3c).

To verify the specificity of real time RT-PCR, we analyzed the expression profiling of two additional genes, ERK2 and CXCR4, and comparing the different experimental condition, the difference in relative mRNAs levels were insignificant both at t1 and t2 (Fig. 3b,c). To monitor CCR5 receptor localization, the presence of CCR5 specific puncta was also analyzed by fluorescence microscopy, at t2. Accordingly with real time RT-PCR data, Fig. 3d shows a statistically significant increase of CCR5, in terms both of the percentage of cells that showed CCR5 puncta into the cytoplasm (p ≤ 0.05) and of the number per cell of CCR5 specific puncta (p ≤ 0.05), upon anti CCR5 Ab Pos stimulation in the cells interfered with β-arrestin1 siRNA. No statistically significant variation was observed from other tested conditions (Fig. 3d). A single cell section from a representative experiment is shown in Fig. 3e. The parallel flow cytometry analysis showed that none of the samples, previously treated with CCR5 Ab Pos, displayed a complete recovery of the CCR5 on the cells surface at t2 (Fig. 3f), actually the CCR5 downregulation in the cells exposed to CCR5 Abs Pos was statistically significant compared to untreated cells (p ≤ 0.01), as expected based on our previous published data^21^.

To complement these findings, we analyzed the involvement of ERK1 in the CCR5 signalosome outcomes by inhibition of ERK1/2; we pretreated the cells for 1 h with U0126, a chemical inhibitor of MAPK phosphorylation, and incubated them with either CCR5 Ab Pos or CCR5 Ab Neg for 48 h (t1). The cells were collected at t1 (48 h) and t2 (48 h, wash, further 24 h) and analyzed by real time RT-PCR. The results demonstrated that the inhibition of ERK1/2 mediated by U0126 did not affect the regulation of CCR5 mRNA expression levels even with CCR5 Ab Pos stimulation (Fig. 3g).

These results, taken together with those obtained with western blot analysis, suggest that β-arrestin2 plays a relevant role as sequester of the complex into the cells and its depletion could address the signalosome to degradation.

### β-arrestin2 is essential for the CCR5 signalosome stabilization

**Figure 4:**
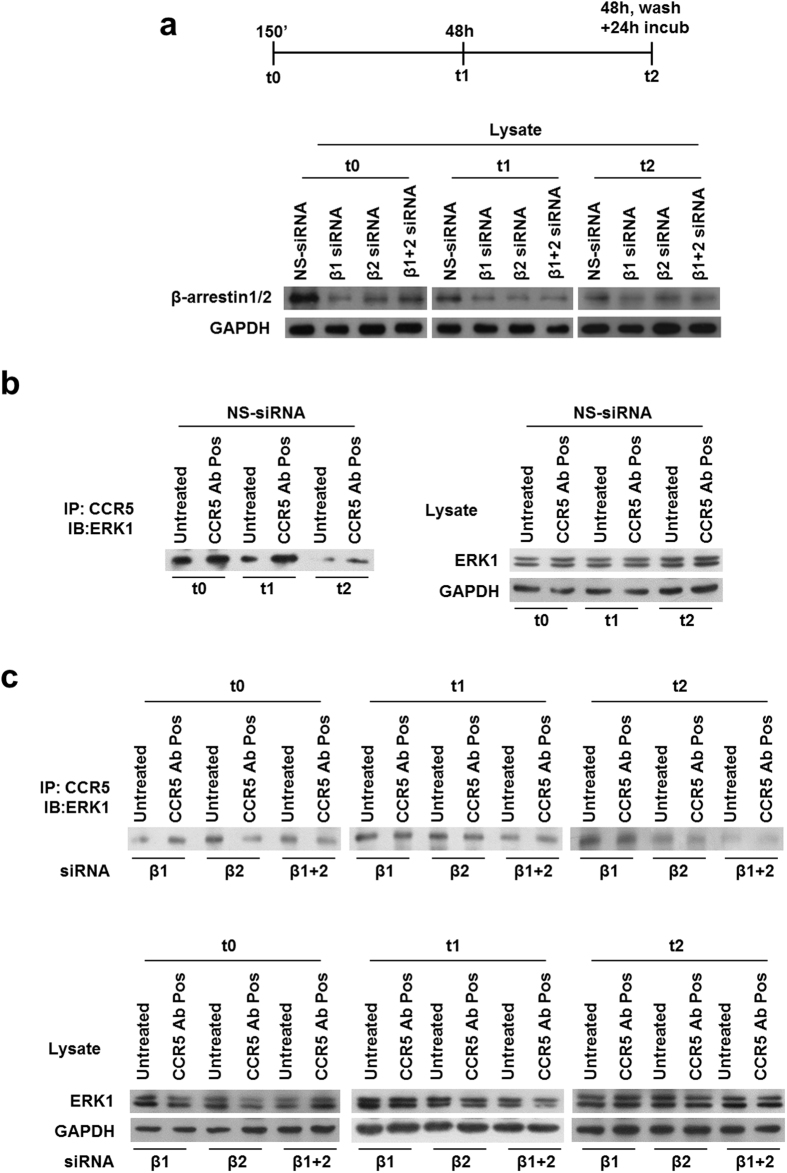
Role of β-arrestin1/2 specific siRNAs in the interaction among CCR5, β-arrestin1/2 and ERK1. (**a**) Schematic representation of kinetics experiment and immunoblot analysis of β-arrestin1/2 and GAPDH (loading control throughout) in whole-R5-SupT1-L23 cell-lysates nucleofected with siRNAs directed to β-arrestin1 (β1), β-arrestin2 (β2) or both and with NS-siRNA (negative control) evaluated at t0 (corresponding to 30′ of CCR5 modulating factors incubation, washing and additional 120′ of cells incubation in medium without stimuli (150′)), t1 (corresponding to 48 h of factors incubation), t2 (corresponding to 48 h of factors incubation, washing and a further 24 h of cells incubation in medium without stimuli). After 5 h of nucleofection, the cells were incubated with CCR5 Ab Pos and appropriate controls (RANTES and CCR5 Ab Neg) and a time course analysis was performed: cells were harvested at all time points and analyzed for β-arrestin1/2 expression by western blotting. (**b**,**c**) After incubation, the cells were lysed and CCR5 immunoprecipitates (IP) were evaluated by western blotting analysis. Immunoblot analysis of total cell lysates was used as a control. One representative of three independent experiments has been shown.

To validate that the depletion of β-arrestins acts as a restriction factor for the CCR5 signalosome formation and β-arrestin2 is really the most important in our model, we transiently nucleofected R5-SupT1-M10 cell line with β-arrestins siRNAs and a relative control and treated the cells with CCR5 Ab Pos as described above. Western blotting analysis displayed that β-arrestin1/2 siRNA duplexes specifically decrease protein expression levels (Fig. 4a). We evaluated the association of endogenous ERK1 protein with the internalized receptor CCR5 by immunoprecipitation assay in the presence or not of specific siRNAs for β-arrestin1 and 2. ERK1 accumulation was evident upon treatment with CCR5 Ab Pos at t0 as well as t1; on the contrary, at t2, the stimulation with CCR5 Ab Pos led to an evident decrease of the amount of ERK1 (Fig. 4b). The immunoblot in Fig. 4c shows that ERK1 had a weak basal level interaction with the receptor and it was robustly recruited to CCR5 following CCR5 Ab Pos stimulation only if β-arrestin1 was silenced, at all time analyzed. Additionally, the depletion of cellular levels of β-arrestin2 occurred in a reduction of the complex formation as shown by the weaker ERK1 binding, especially at t0 and t1 (Fig. 4c). These data suggest not only that the receptor under stimulation with anti-CCR5 Ab Pos utilizes a β-arrestin2-dependent endocytic mechanism but also that β-arrestin2 is necessary for the signalosome stabilization.

### Cycloheximide treatment drives the *de novo* synthesis of β-arrestin2 only

**Figure 5:**
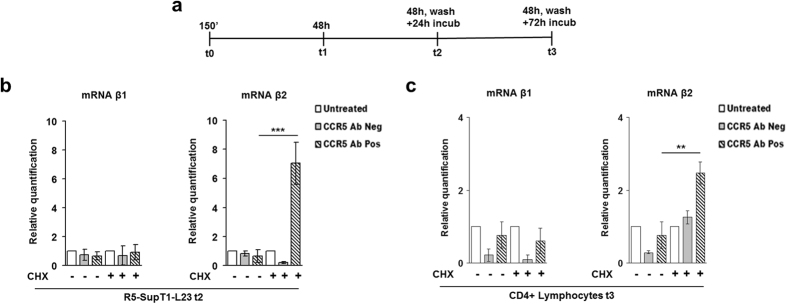
Effects of CHX treatment on β-arrestin1/2 de novo synthesis. (**a**) Schematic representation of kinetics experiment: the cells were pretreated for 1 h with cycloheximide (CHX) and then incubated, or not, with CCR5 Ab Pos and CCR5 Ab Neg. The cells were harvested at t0 (corresponding to 30′ of CCR5 modulating factors incubation, washing and additional 120′ of cells incubation in medium without stimuli (150′)), t1 (corresponding to 48 h of factors incubation), t2 (corresponding to 48 h of factors incubation, washing and a further 24 h of cells incubation in medium without stimuli) and t3 (corresponding to 48 h of factors incubation, washing and a further 72 h of cells incubation in medium without stimuli). (**b**) Real time RT-PCR analysis of β-arrestin1 (β1) and β-arrestin2 (β2), in R5-SupT1-L23 cell line treated with the CHX inhibitor and stimulated with CCR5 Ab Pos and the appropriate control, compared to the untreated cells, at t2. (**c**) Real time RT-PCR analysis of β-arrestin1 (β1) and β-arrestin2 (β2), in CD4+ T lymphocytes treated with the CHX inhibitor and stimulated with CCR5 Ab Pos and the appropriate control, compared to the untreated cells, at t3. Bar graphs represented mean ± SD of three independent experiments. Student’s t-test was performed and p values are shown. *p ≤ 0.05, **p ≤ 0.01, ***p ≤ 0.001.

We have recently published that CCR5 Ab Pos induce the *de novo* synthesis of ERK1-mediated CCR5 by the aid of treatment with cycloheximide (CHX)^21^. Here we repeated the same experiment, both in R5-SupT1-L23 cell line and in human CD4+ T lymphocytes, to corroborate the role of β-arrestin2 in the CCR5 signalosome. Briefly, the cells were accordingly treated (or not) with the translational inhibitor cycloheximide (CHX) and stimulated (or not) with CCR5 Ab Pos and their control. The cells were collected at t1 (48 h), t2 (48 h, wash, further 24 h) and t3 (48 h, wash, further 72 h), as described in Fig. 5a. We used the real time RT-PCR analysis to ascertain whether the restoration of protein synthesis at t2 meant a *de novo* synthesis of the genes involved in the CCR5 signalosome under CCR5 Ab Pos treatment. All results already obtained and published in Venuti *et al*.^21^ were confirmed (data not shown); in addition, a statistically significant up-regulation in gene expressions was shown with β-arrestin2 (p ≤ 0.001), and not with β-arrestin1, in R5-SupT1-L23 at t2 (Fig. 5b). When CD4+ T lymphocytes were analyzed a statistically significant increased gene expression was shown with β-arrestin2 only and exclusively at t3 (p ≤ 0.05) (Fig. 5c). Taken together, these data suggest that all the active proteins of the signalosome are newly synthetized rather than recycled.

## Discussion

The signaling activity of CCR5 is partially controlled by internalization, recycling, and/or degradation, and it is not fully understood yet. Natural antibodies recognizing the ECL1 domain of CCR5 have been found both in “naturally vaccinated” subjects, i.e. multiple exposed to HIV but seronegative subjects defined as ESN^32^, and in Long Term Non Progressors subjects (LTNP)^33^,^25^; in both cases they have been associated to total (in ESN) or partial (in LTNP) resistance to HIV infection as the mechanism of action is a full and long lasting CCR5 internalization, which blocked HIV infection in either CD4+ T lymphocytes or transcytosis (which mimics mucosal transmission)^34^, and this mechanism is different from that reported for all other CCR5 specific molecules^20^. There are several differences between the present study and previous published studies based on the use of ligands that explain our different findings. The major difference is that we analyzed antibody-CCR5 internalization through binding to the first loop of CCR5, whereas previous works described the activity of CCR5-agonist–mediated endocytosis, which specifically bind the second external loop of CCR5. We believe that different properties of each CCR5 extramembrane region, as revealed by the use of monoclonal antibodies to the N-terminus and to the second loop of CCR5, differentially modulate and influence receptor activity^35^. Moreover, in an experimental BalB/c mouse model, we recently found that ECL1 and ECL2 but not N-terminus antisera induced nearly complete long-lasting CCR5 downregulation of the receptor, thus suggesting that epitopes within each CCR5 extramembrane regions may be responsible of the different trafficking^36^. Indeed, we previously demonstrated that the substitution of some aminoacids within ECL1 elicits antibodies, which do not induce the long lasting internalization of CCR5^37^, thus confirming that the epitope is extremely relevant to drive the different trafficking pathway. On the other hand different ligands of the same GPGR can elicit different phosphorylation pathways, which could play an important role in the interaction with β-arrestins^38^.

Another huge difference between natural antibodies activity and ligands, such as Rantes, is that the ligands induce a classical short-term kinetics of CCR5 internalization with transient activation of β-arrestins and rapidly recycling or degradation on the cell surface; on the contrary, natural Abs to ECL1 of CCR5 show a peculiar long-lasting kinetics of CCR5 internalization^25^ with the involvement of an ERK1-based pathway, as previously reported^21^. In particular, the time course analysis performed in our previous work by flow cytometry shows as the CCR5 downregulation is achieved after 48 h of treatment with CCR5-specific Ig, whereas an intermediate value is obtained after 24 h of incubation^25^. In contrast, CCR5 modulation is not evident after 1 h of treatment^25^ but it starts after 150′, as recently reported^21^. Moreover, in an experimental BalB/c mouse model, we induced and reproduced the specificities found in ESN and in LTNP with a similar kinetics. Indeed mouse Abs to ECL1 of CCR5 *in vivo* induce CCR5 internalization and reexpression, following a very slow kinetics that is gradually recovered 4 weeks after immunization^39^. This finding is potentially relevant for the design of appropriate microbicide candidates that may prevent HIV infection for several days after specific application of a compound.

An exciting and unexpected finding of this work is the translation of a Class A trafficking pattern, normally used by CCR5 for its internalization, to a very long lasting Class B type upon stimulation with natural human antibodies to CCR5^1^,^12^. Bönsch and colleagues have recently demonstrated the different capability of two Rantes analogous (PSC and 5P14) to elicit the formation of durable complexes between CCR5 and β-Arrestin1. In detail, PSC-Rantes induces a long-duration of recruitment of β-Arrestin1 to CCR5 instead of 5P14-Rantes, which provides a transient recruitment. Of note, the experiments have been performed and the results evaluated only at short time (50′)^38^. Thus, the fate of internalized CCR5 can be determined by the modified ligand that engaged it, suggesting that the duration of ligand-induced receptor-arrestin association is likely to play a key role in the sorting mechanism^38^. In our model, the anti-CCR5 antibodies induce a long-lasting down-regulation of CCR5 accomplished at 48 h with consequent *de novo* CCR5 synthesis, as we extensively reported in 21. Our results not only demonstrate a switch in the two β-arrestins activity, which can act in a complementary way, but also the exposure of natural antibodies leads differences in CCR5 signaling, which drives ligand- directed post-endocytic sorting in a very long lasting Class B trafficking. Other post-translational modifications may also contribute to the different trafficking mechanism, for example it has been shown that the trafficking itinerary of another chemokine receptor, CCR7, is affected by the extent to which it is ubiquitinylated^40^. As far as we know, for the first time our work demonstrates such long lasting Class B pathway.

We cannot exclude that the reason of switching from class A to class B could be due to antibodies which are bivalent and they could result in crosslinking of CCR5 receptor and/or in a better affinity, although we did not see any difference in term of long lasting internalization between IgG and IgA^25^,^33^, thus it is unlikely that the different pattern could be to the nature of antibodies vs ligands.

Our studies have been performed in T cells, such as R5-SupT1-transducted cell lines and in CD4+ T lymphocytes, in which, based on our knowledge, we have described for the first time a mechanism where anti CCR5 Abs induce an ERK1-mediated CCR5-negative phenotype by the aid of β-arrestin2; otherwise, we cannot exclude that this mechanism is specific for T cells only.

As several studies have shown a relevant role of β-arrestins in the internalization process of GPCRs^3^,^4^,^5^,^6^,^27^, here we demonstrate that, upon stimulation with anti-CCR5 Abs but not with Rantes, β-arrestins form tight and steady complexes with both the receptor and ERK1 protein (Fig. 1). Supporting our results, recent published data have shown that β-arrestins play a relevant role in the sequestration of GPCRs into the cytoplasm, due to the formation of stable complexes between GPCRs and β-arrestins themselves. The stability of activated GPCR-β-arrestin complexes and their cotrafficking to endosomes are defined by both the nature of the phosphorylation barcode on cytoplasmic domains of GPCRs and the ubiquitination status of β-arrestin^1^,^12^. The endosomes containing complexes between activated GPCR, activated and ubiquitinated β-arrestin and phosphorilated ERK are named as “signalosome”^1^,^9^. Moreover, these cellular processes could be accomplished by either β-arrestin1 or β-arrestin2, depending on the receptors^15^: for the V2R (vasopressin receptor V2), this function is exclusively carried out by β-arrestin2 isoform, while the isoform 1 has an inhibitory role^41^,^42^; on the other hand, both β-arrestin isoforms are important for PAR-2 (protease-activated receptor-2) modulation mechanism^43^,^44^. Based on these, we established which isoform is the most important in our model by depleting cellular levels of β-arrestin1 or -2 or both with siRNAs specific to each isoform. In our experiments, we obtained an unequivocal correlation between the absence of functional β-arrestin2 protein and the reduction of cytosolic accumulation of both CCR5 and ERK1 proteins in the presence of CCR5 Abs, during early event (t0) as well as when the internalization reached the maximum level (t1) (Fig. 2).

Surprisingly, the silencing of β-arrestin1 only, upon treatment with CCR5 Ab Pos, showed a statistically significant accumulation of CCR5 signalosome proteins at t2, that is 24 h post washing from all stimuli. Data obtained by real time RT-PCR showed that these formations of stable CCR5 signalosome complexes, in the cells interfered with β-arrestin1 and stimulated with CCR5 Ab Pos, could be due to a shift in mRNA synthesis of β-arrestin2 (Fig. 3). Conversely, the abrogation of β-arrestin2 isoform expression, in the cells exposed to CCR5 Ab Pos, was responsible for the statistically significant *de novo* synthesis of all the major CCR5 signalosome proteins, at t1 (Fig. 3). Considering the capability of modified Rantes to induce CCR5 and β-arrestin1 complexes in a stable short term kinetics (50′)^38^, we can assume that both β-arrestins can be differentially activated under ligand stimulation. For this reason our hypothesis is that the absence of functional β-arrestin2 does not allow the ubiquitination of β-arrestin2 itself and prevents the cytoplasmic accumulation of both CCR5 and ERK1 proteins (Fig. 2). In addition, although we have no direct evidence of CCR5 degradation, in our previous work^21^, we blocked protein synthesis, with the translational inhibitor cycloheximide (CHX), to evaluate the respective rates of recycling and CCR5 receptor re-synthesis. Our data indicate that the restoration of protein synthesis at t2 for R5-SupT1-L23 cells in CHX experiments clearly showed a new synthesis of CCR5 mRNA at a cytoplasmic level after CCR5 Ab Pos stimulation followed by the re-expression of CCR5 on the membrane of R5-SupT1-L23 cells. Based on this evidence we assume that the prolonged sequestration of CCR5 into the cytoplasm can lead to the final destiny, which is degradation of the receptor. Furthermore, under physiological conditions the restoration of CCR5 surface expression in R5-SupT1-L23 was clearly seen 8 days after wash^21^. All together these data seem to indicate that, only when β-arrestin2 is knocked-down, does CCR5 form transient complexes with the other components of the signalosome (ERK1 and β-arrestin2 itself), which are rapidly degraded (Fig. 3). These data were further validated by flow cytometry and immunofluorescence analyses, where none of the samples previously treated with CCR5 Ab Pos displayed a complete recovery of the CCR5 on the cell surface at t2, for all silencing conditions tested; on the contrary, the cells interfered with β-arrestin1 siRNA and treated with anti CCR5 Abs revealed an accumulation of CCR5 into the cytoplasm, in accordance with those obtained using western blot and real time RT-PCR (Fig. 3). When the analyses were performed at t1, a complete CCR5 downregulation upon stimulation with CCR5 Ab Pos was observed as expected (data not shown). Unfortunately, due to the nucleofection technique, at t0 was performed immunofluorescence analysis only and we found a significant difference in the reduction on the accumulation of cytoplasmic CCR5 upon stimulation of CCR5 Ab Pos in absence of β-arrestin2 measured by percentage of punctate cells (NS-siRNA: 66%, siRNA β1: 60%, siRNA β2: 40%, siRNA β1 + 2: 45%) and of number of CCR5 puncta/cell (NS-siRNA: 5, siRNA β1: 4, siRNA β2: 2.3, siRNA β1 + 2: 2.3). These data were further confirmed in the western blot analyses showed in Fig. 2.

Moreover, the data on the interaction between the major players of the CCR5 signalosome, after depleting cellular levels of either β-arrestin1 or β-arrestin2 and consequent treatment with anti CCR5 Abs, underlined that CCR5-ERK1 sequestration was stably generated only when β-arrestin2 was functional (Fig. 4); these results indicate that β-arrestin2 is not only a scaffold protein to ERK1 activation but it also functions as an indispensable player for the formation and the long lasting stabilization of the said complexes. Finally, outcomes obtained by chemical inhibition of protein synthesis clearly showed that the major proteins of the CCR5 signalosome, including β-arrestin2, were newly synthetized rather than recycled (Fig. 5). Overall data obtained at t2 (western blot, real time RT-PCR and immunoprecipitation) could be the result of a combination of specific effects of anti CCR5 Abs on modulation of the receptor and physiological cellular turnover.

**Figure 6:**
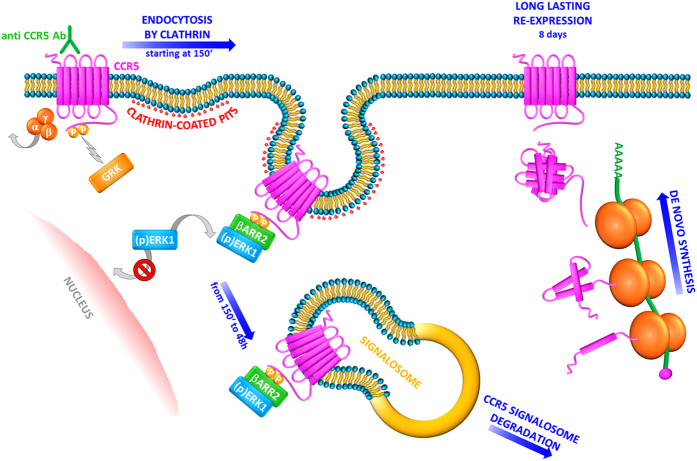
Class B CCR5 trafficking pathway triggered by CCR5 Ab Pos in T cells. Upon stimulation with CCR5 Ab Pos, the CCR5 receptor associates with G protein and G protein-coupled receptor kinases (GRKs) induce receptor phosphorylation. β-arrestin1/2 can initiate desensitization, thus contributing to the internalization of CCR5 by clathrin-coated pits. In particular, β-arrestin2 accumulates in protein complexes with activated CCR5 and leads to activation and retention in the cytoplasm of MAP kinase ERK1. These events contribute to the formation of a CCR5 signalosome with β-arrestin2 and ERK1 into the cytosol, which remains stable from 150′ up to 48 h. The signalosome could be targeted for degradation with consequent *de novo* synthesis of the proteins complex (CCR5, β-arrestin2, ERK1). As a consequence, CCR5 reappears on the cell surface with long lasting kinetics (8 days).

To summarize, it is well known that GPCRs, following activation, are desensitized and internalized. After endocytosis, the GPCR superfamily can be divided into receptors that are recycled, rapidly or more slowly, to the cell surface in a resensitized form and those are targeted for degradation^45^,^46^,^47^. CCR5 belongs to the group of GPCRs that is recycled after desensitization^19^: CCR5 internalized by natural ligands is transported to the trans-Golgi network (TGN) via the endosome recycling compartment (ERC)^48^ and then returns to the cell surface when the resensitization process is complete^19^,^48^. Nevertheless, a few examples of ligand-driven post-endocytic of GPCRs have been already described^49^,^50^,^51^. Interestingly, our results indicate that the stable recruitment of β-arrestin2 to CCR5 is the key directing post-endocytic trafficking; in particular, anti CCR5 Abs trigger a long lasting internalization of the receptor by inducing the tight interaction between CCR5 and β-arrestin2, which also promote the cytoplasmic retention of ERK1 protein, and lead to the formation of the CCR5 signalosome, which persists into the cells at least 48 h. The signalosome could be targeted for degradation with consequent *de novo* synthesis of the proteins complex (CCR5, β-arrestin2, ERK1). As a consequence, CCR5 reappears on the cell surface with long lasting kinetics (8 days) (Fig. 6).

Further investigations about endocytic pathways involved by anti CCR5 Abs, in T cells, are necessary to better understand the cellular machinery involved in this newly identified inhibitory pathway for CCR5 cell surface expression that may prevent HIV infection. These findings could also help to find novel therapeutic tools as CCR5 plays a role in the distribution of effector cells to sites of microbial infection where CCR5 contributes to microbial control and/or elimination^17^, in the regulation T cell function in autoimmune diseases, including multiple sclerosis, rheumatoid arthritis, and type 1 diabetes^52^ and in tumorigenesis^53^,^54^.

## Methods

### Cell lines

SupT1 cell line (NIBSC, CFAR, UK) was maintained in RPMI 1640 (Lonza, Belgium) supplemented with 10% FCS (Lonza), 2 mM L-glutamine, 100 U/mL penicillin and 100 U/mL streptomycin. R5-SupT1-transducted cell lines are CCR5-expressing cell lines referred to as SupT1-R5 clones L23 (low expression of CCR5) and M10 (medium expression of CCR5). The clones were obtained by engineered SupT1 cells and kindly provided by H. Garg. L23 and M10 clones were propagated in complete medium as described above and supplemented with 3 μg/mL of blasticidin (Calbiochem, Germany). Cell lines were cultured at 37 °C in a 5% CO_2_ incubator.

### Reagents

Anti-CKR-5 (D6), anti-p-CKR-5(E11/19) that it is recommended for the detection of Ser349 phosphorylated (a specific GRK phosphorylation site)^16^, anti-GAPDH MAbs and anti-ERK1 (C-16), anti-p-ERK1/2 (Thr 202/Tyr 204) polyclonal antibodies were purchased from Santa Cruz Biotechnology (Santa Cruz, CA). β-Arrestin1/2 (D24H9) Rabbit monoclonal antibody was purchased from Cell Signaling Technology (Beverly, MA) which detects endogenous level of total β-arrestin1 and β-arrestin2 proteins showing a single band only at 50 KDa as reported in the datasheet. PE Mouse anti-Human CD195 (2D7), and PE Mouse IgGs Isotype Control were purchased from BD-Pharmingen (San Diego, CA). Secondary HRP-conjugated goat anti-mouse IgG and goat anti-rabbit IgG were purchased from Millipore; secondary FITC-conjugated goat anti-mouse IgG was from ICN Biomedicals (Aurora, OH). Secondary HRP-conjugated anti-mouse IgGVeriBlot and anti-rabbit IgGVeriBlot for IP were from Abcam (UK). The pool of serum samples from 5 LTNP positive for anti-CCR5 natural antibodies (CCR5 Ab Pos) were: LTNP no. 21, LTNP no. 20, LTNP no. 11, LTNP no. 4, and LTNP no. 22, all of which had been characterized previously^21^,^25^. The pool of serum samples used as negative control were taken from LTNP negative for anti-CCR5 antibodies (CCR5 Ab Neg), as previously described^21^,^25^.

### Ethics Statement

The Institutional review board named “Comitato Etico della Fondazione San Raffaele del Monte Tabor, Milan, Italy” approved the investigations. Protocol no 95/DG. All subject provided the written informed consent and all methods were performed in accordance with the relevant Italian guidelines and regulations.

### Purification of human CD4+ T lymphocytes

Human peripheral blood mononuclear cells (PBMCs) from 3 healthy donors were isolated with Ficoll-Hypaque (Pharmacia, Uppsala, Sweden). CD4+ cells were purified from resting PBMC by immune adsorption to anti-CD4 magnetic beads (Miltenyi Biotech, Italy). Purified CD4+ T lymphocytes were stimulated for 16 h in RPMI 1640 supplemented with 10% FCS (Lonza), 2 mM L-glutamine, 100 U/mL penicillin and 100 U/mL streptomycin in the presence of recombinant interleukin 2 (IL-2) (100 U/ml; Amersham, Buckinghamshire, United Kingdom); such lymphocytes were then cultured at 37 °C in a 5% CO_2_ incubator. All donors were wild type for CCR5, none of them carrying the Δ32 mutation.

### CCR5 expression and internalization assay

R5-SupT1-L23 cells or CD4+ T lymphocytes were exposed to CCR5 Ab Pos (1/30), and to 2 μg/mL RANTES (R&D Systems, MN) as a positive control, at 37 °C for 30′. Serum dilution and RANTES concentration have been chosen based on previously titration experiments; in particular serum samples have been tested at 1:10, 1:30 and 1:50 and 1:30 was the first not toxic dilution compared to serum samples negative for anti CCR5 Abs. RANTES was tested at 5, 2 and 0.5 μg/mL and 2 μg/mL was the minimum concentration able to bind CCR5 and induce the highest percentage of receptor internalization (data not shown). The cells were then washed and incubated for an additional 120′ (t0). To obtain a complete down-regulation of the receptor, the cells were incubated with CCR5 Ab Pos at 37 °C for 48 h (t1)^25^. CCR5 Ab Neg was used as a negative control^25^. When indicated, the cells were pre-treated for 1 h with U0126 (a specific inhibitor of ERK pathway) (5 μg/mL) and with 2.5 μg/mL cycloheximide (CHX) (Sigma Aldrich) to inhibit protein synthesis. As U0126 has been reconstituted in DMSO, the same percentage of DMSO diluted in RPMI was used as a negative control (data not shown). After 48 h of CHX treatment, R5-SupT1-L23 and CD4+ T lymphocytes were washed and incubated in complete medium for an additional 24 h (t2) and 72 h (t3), respectively. Immunofluorescence was performed to detect cytoplasmic CCR5. The samples were collected, layered on slides treated with polylysine, and fixed in 4% paraformaldehyde in PBS (pH 7.4) for 15′ at rt, and permeabilized with 0.1% Triton X-100 in PBS for 15′. Cells were then incubated with anti-CKR-5 (D6) primary antibody for 45′ at 4 °C, followed by incubation with FITC-conjugated secondary antibody. Samples were analyzed on a Leica DMRE fluorescence microscope. In some cases, flow cytometry was performed as well to detect cell surface expression of CCR5. Treated cells were collected, washed and incubated with the specific antibody against CCR5, anti-CD195, for 45′ at 4 °C. Sample data were acquired using FACScalibur (BD) and analyzed with FlowJo Software (TreeStar, San Carlos, CA). For each analysis, 10,000 events were acquired and gated on CCR5 expression and side scatter properties. Samples were first run using isotype control for color compensation.

### Western blotting

Protein lysates were collected in RIPA lysis buffer (50 mM Tris-HCl, pH 7.5, 10 mM MgCl_2_, 150 mM NaCl, 0.5% sodium deoxycholate, 1% Nonidet P-40) supplemented with complete protease inhibitor cocktail (Roche). Samples were resolved by SDS–PAGE and transferred to nitrocellulose membranes (Amersham Pharmacia Biotech AB). The membranes were blocked in 5% non-fat milk and incubated overnight at 4 °C with the appropriate primary antibody. The membranes were then probed with HRP-tagged secondary antibodies at room temperature for 1 h. Immunoreactive proteins were visualized by means of the ECL method (Millipore). Comparative analysis of the bands was performed by quantitative densitometry and concomitant use of the Tina software (version 2.10, Raytest, Straubenhardt, Germany). The normalization was done per each band based on the density of GAPDH signals per each line.

### Immunoprecipitation

The cells were treated in accordance with the experimental procedure for CCR5 internalization assay. At t0, t1 and t2, cells were washed twice in PBS and lysed with cold lysis buffer (20 mM Tris-HCl, pH 8, 1 mM EDTA, 200 mM NaCl, 1% Nonidet P-40, 2 mM DTT, 0.1 mM Na_3_VO_4_, 10 mM NaF, 0.1 μg/mL Protease Inhibitors). The supernatants were collected and precleared with 50% of protein-A slurry for 16 h. Immunoprecipitations were performed with 5 μL of the indicated antibodies pre-adsorbed on protein A-Sepharose beads (Amersham Pharmacia Biotech AB) for 2 h at 4 °C^21^,^55^. In particular, we used a monoclonal antibody anti-CKR-5 (D6), which specifically recognizes ECL2 of CCR5, thus it does not interfere with the activity of CCR5 Ab Pos that are specific for ECL1 domain of CCR5 only (data not shown). After overnight incubation with the extracts, complexate-beads were resolved by SDS–PAGE and transferred to nitrocellulose membranes (Amersham). Immunoblotting was performed with either ERK1 or β-arrestin1/2 antibodies and immunoreactivity was revealed by means of secondary antibodies specific for IP (Abcam). Immunoreactive proteins were visualized by means of the ECL method (Millipore).

### Real-time RT–PCR

Total RNA was isolated from each sample with a QiagenRNeasy mini-kit (Qiagen, CA). Subsequently, RNA was used for reverse transcription (RT) to cDNA by means of SuperScript^®^ III First-Strand Synthesis System (Invitrogen, CA) in accordance with the manufacturer′s instructions. Gene transcript levels were analyzed with SYBR^®^-Green-PCR Master Mix on an AB 7900HT real-time system (Applied Biosystems, CA). The termal profile was 50 °C for 2′; 95 °C for 10′; 40 cycles at 95 °C for 15”; 60 °C for 1′. GAPDH amplification was used to normalize the RNA content of the corresponding transcripts analyzed.

Commercial primers were Hs_MAPK3_1_SG (ERK1), Hs_MAPK1_1_SG (ERK2), Hs_ARRB1_1_SG (β-arrestin1), Hs-ARRB2_1_SG (β-arrestin2) QuantiTect^®^ Primer Assay (Qiagen). Primer sequences were: 5′-ACCAGATCTCAAAAAGAAGGTCT-3′ (CCR5-forward); 5′-CATGATGGTGAAGATAAGCCTCA-3′ (CCR5-reverse); 5′-TGGAACACAACCACCCACAA-3′ (CXCR4-forward); 5′-CCCTGCCCTCCTGCTGACTA-3′ (CXCR4-reverse); 5′-CCATGGAGAAGGCTGGGG-3′ (GAPDH-forward); 5′-CAAAGTTGTCATGGATGACC-3′ (GAPDH-reverse). Relative expression was calculated with the ΔΔCt standardization method.

### siRNA nucleofection

Chemically synthesized, double stranded siRNAs were purchased from Eurofins Biolab Italia. siRNA sequences targeting human β-arrestin1 and 2 and GAPDH (NSsiRNA) were already described^15^. Briefly, 300 nM siCCR5 or were used to nucleofect 2 × 10^6^ cells by means of an Amaxa Nucleofector Device (Lonza) in accordance with the manufacturer’s instructions. The cells were then seeded onto 24 multi-well plates for 5 h and treated in accordance with the experimental procedure for CCR5 internalization assay.

### Statistical analysis

The two-tailed Student’s t-test was used and the data were analysed by Prism version 5.0a (GraphPad Software, La Jolla, California, USA). Figures 2, 3 and 5 show p values: *stands for p ≤ 0.05, **stands for p ≤ 0.01 and ***stands for p ≤ 0.001.

## Additional Information

**How to cite this article**: Venuti, A. *et al*. Class B β-arrestin2-dependent CCR5 signalosome retention with natural antibodies to CCR5. *Sci. Rep.* **6**, 39382; doi: 10.1038/srep39382 (2016).

**Publisher's note:** Springer Nature remains neutral with regard to jurisdictional claims in published maps and institutional affiliations.
